# Nurse Leaders’ Perceptions and Practices of E‐Leadership: A Qualitative Study

**DOI:** 10.1155/jonm/2583936

**Published:** 2026-01-08

**Authors:** Vanesa Numanovic, Harri Jalonen, Juha Lindell

**Affiliations:** ^1^ School of Management, Social and Health Management, University of Vaasa, Vaasa, Finland, uva.fi; ^2^ Head and Neck Center, University of Helsinki and Helsinki University Hospital, Helsinki, Finland, helsinki.fi

**Keywords:** leadership, nurse administrators, nursing, organizational innovation, remote consultation

## Abstract

**Introduction:**

While research on nursing leadership has grown, empirical studies specifically addressing e‐leadership in nursing remain scarce. Digitalization offers opportunities to enhance efficiency and flexibility but also necessitates careful management of interpersonal relationships and staff well‐being. E‐leadership in nursing requires strong interpersonal competencies, organizational support, targeted training, and clear policies, along with a balanced leadership approach. Notably, there is a lack of research on the state of e‐leadership in nursing in the postpandemic context.

**Aim:**

This study aims to explore the current state of Finnish nurse leaders’ experiences with e‐leadership.

**Methods:**

A qualitative research design was employed, utilizing individual thematic interviews and inductive content analysis. Fourteen individual interviews were conducted. The Standards for Reporting Qualitative Research (SRQR) checklist guided the reporting process.

**Results:**

The analysis yielded three main categories: (1) E‐leadership in nursing is here to stay, (2) a lack of consistent e‐leadership practices in nursing, and (3) e‐leadership presents challenges for both nurse leaders and staff. Although e‐leadership remains under development and lacks clearly defined structures, both nurse leaders and staff demonstrate a willingness to adopt and implement this leadership model. However, organizational support has been insufficient, often leaving nurse leaders to independently develop solutions to the challenges they face.

**Conclusion:**

This study reinforces previous findings on e‐leadership in nursing while offering new insights into its current state. E‐leadership is a dynamic and evolving practice that requires a delicate balance between digital efficiency and human connection. Despite its challenges, e‐leadership presents significant opportunities for the nursing profession, including enhanced flexibility and improved work–life balance.

**Implications for Nursing Management:**

The findings can inform the development of practical guidelines for implementing e‐leadership in nursing and healthcare settings.

## 1. Introduction

E‐leadership is becoming increasingly relevant in healthcare settings, particularly following the COVID‐19 pandemic, which accelerated the adoption of remote work across sectors, including nursing [1]. While research on e‐leadership began in the 2000s and has expanded within business, management, and ICT disciplines [2], studies focusing specifically on healthcare contexts remain relatively scarce. However, healthcare leaders face unique challenges in digital environments. Nurse leaders (NLs) must utilize technology to ensure the flow of information, build trust, and balance digital and traditional forms of communication [3]. E‐leadership is beset with issues around communication [4], trust‐building [5], lack of interpersonal contact [1], and feelings of isolation [6]. NLs and nurses play a central role in healthcare digitalization. Effective e‐leadership involves building trust and establishing a virtual presence [7].

An integrative review by Terkamo‐Moisio et al. [8] identified a lack of organizational support as a key barrier to effective e‐leadership in healthcare teams. Moreover, there are no universally accepted guidelines for e‐leadership in nursing [9], and NLs often report feeling overwhelmed by technological demands [10, 11]. The work of Avolio et al. [12] cautions that e‐leadership should not be viewed merely as a digital extension of traditional leadership, but rather as a distinct, evolving phenomenon that warrants context‐sensitive understanding and support structures.

E‐leadership is defined in various ways, typically focusing on social influence processes, conditions, or competencies [13]. A widely accepted definition describes e‐leadership as a “social influence process mediated by advanced information technology (AIT) to produce a change in attitudes, feelings, thinking, behavior, and/or performance with individuals, groups, and/or organizations” [14]. That definition was later refined to emphasize the context in which e‐leadership occurs ([12], p. 107). Aspects typical of e‐leadership include geographical distance between leaders and employees and communication via technology solutions [2].

Digitalization can enhance efficiency and flexibility; it also requires careful management of interpersonal relationships and well‐being. In the nursing context, e‐leadership requires strong interpersonal skills to maintain trust and connections and organizational support to provide clear structures, training, and policies. E‐leadership also thrives on balanced leadership approaches, which might involve hybrid models combining in‐person and virtual leadership. Ongoing research and organizational support are essential to optimize e‐leadership practices in nursing [15]. The effects of the COVID‐19 pandemic included healthcare organizations being forced to rapidly adopt e‐leadership practices [9, 16]. That shift did prompt some empirical research on e‐leadership in nursing; however, recent studies on the state of e‐leadership in nursing postpandemic are scarce. Kiljunen et al. [17] reported that e‐leadership in nursing remains underresearched and call for more empirical studies, particularly those with a practical perspective. We concur that the topic is both timely and critical for future development in healthcare leadership. The present study aims to describe Finnish NLs’ current perceptions of e‐leadership in their profession. Our research questions were as follows:

1. How do NLs perceive and practice e‐leadership in the context of specialized healthcare services?
2. What organizational procedures and practical approaches support e‐leadership among NLs in specialized healthcare settings?

First, this article contributes by adding to the relatively sparse research on e‐leadership in nursing. The second contribution lies in highlighting how contextual factors in healthcare shape leadership practices and perceptions of e‐leadership in nursing. The article’s main managerial contribution is the recommendation that leadership development programs address the complexities involved in leading care professionals remotely.

## 2. Methods

### 2.1. Study Design

The limited information available on e‐leadership in nursing prompted a qualitative design utilizing individual thematic interviews [18] and inductive content analysis [19]. A qualitative approach was chosen to explore the NLs’ experiences regarding e‐leadership from their perspectives. The thematic interview guide was developed based on previous literature on e‐leadership. The Standards for Reporting Qualitative Research (SRQR) checklist (see Supporting Information (available here)) guided the reporting of the study [20].

Trustworthiness in this qualitative study was ensured through strategies such as maintaining an audit trail and providing thick descriptions to support credibility, dependability, and transferability. To enhance analysis credibility, we included original quotations. We strive to adhere to ethical scientific practices at every stage of the research process [21].

### 2.2. Participants and Recruitment

Purposeful sampling was employed [22]. Fourteen eligible individuals who were currently working as frontline NLs and had experience in e‐leadership agreed to participate (Table 1). The hospital provided a list of all nurse managers (*N* = 155), to whom invitation emails were sent. A total of 29 responses were received. Of these, 13 did not meet the inclusion criteria, primarily because the respondents were not responsible for nursing staff but for other healthcare professionals. In this hospital, the title “nurse manager” may also refer to leaders of other professional groups, such as physiotherapists. The remaining nonparticipation was due to lack of response following initial contact. After conducting 11 interviews, we observed signs of data saturation. Three additional interviews had already been scheduled, which we proceeded to conduct. Following these interviews, we were confident that data saturation had been achieved. Written informed consent was obtained from all participants.

**Table 1:** Overview of inclusion and exclusion criteria.

	*n*
Total responses	29
Inclusion criteria:	16
1. Front line nurse leader, e.g., nurse manager	
2. Experience in nursing e‐leadership	
3. Sufficient spoken proficiency in Finnish	
Exclusion criteria:	13
1. Leader of other healthcare professionals	
2. No experience in nursing e‐leadership	
Excluded for other reasons	2
Final number of included participants	14

### 2.3. Data Collection

Prior to data collection, a research permit for the study was obtained from the healthcare organization. First, we conducted a pilot study to ensure that the interview questions were clear and relevant to the study, and to refine our interview skills [23]. The pilot interviews were excluded from the data [24]. We followed the recommendations for qualitative research by Elo et al. [19]. We interviewed the participants between October and December 2024 either in person or with the Microsoft Teams application. All interviews were conducted by the first author, a doctoral researcher in social and health management, utilizing a consistent thematic guide [18, 25] (see interview guide in Supporting Information (available here)). Participants’ characteristics were collected at the beginning of the interviews. Interviews were audio‐recorded and ranged from 35 to 85 min, with an average duration of 71 min. The outcome was 435 min of recorded data.

### 2.4. Data Analysis

All interviews were transcribed verbatim, resulting in 258 pages of text (font Calibri 11 pt spacing 1.0). The data were then subjected to content analysis [19, 26] using NVivo15 software [27]. Initially, meaning units were identified and subsequently condensed into codes. These codes were then categorized based on their semantic relationships to form subcategories. Table 2 illustrates one example of the analysis process.

**Table 2:** An example of the analysis process.

Quotation	Code	Subcategory	Category	Main category
*I have perhaps noticed that trust takes time. I must say that time is on our side.*	Building trust	Trust as the cornerstone of e‐leadership	Key factors for success in e‐leadership in nursing	E‐leadership is a challenge for nurse leaders and staff
*Of course, if it turns out that this person is not actually doing the work for some reason, then that would be a worse situation.*	Loosing trust			
*Or if they have even arranged it themselves, then we will start to address it. I don’t know if we talk about it like, “I suspect you don’t work there.” I don’t really believe that, or it’s somehow taboo.*	Talking about trust			
*So, it is a challenge of e-leadership; how do you measure the employee’s work to ensure that the distribution of tasks is fair?*	Measure and monitor work remotely			
*In my opinion, the most crucial thing is that if I didn’t trust that the employees were doing their jobs, then e-leadership wouldn’t work at all.*	Is based on trust			
*Self-direction is not very easy for all employees.*	Self‐direction	E‐leadership and remote work require self‐direction and motivation.		
*Leadership of one’s own work is needed in this.*	Self‐leadership			
*If the staff is motivated and well-being, they should not need to be monitored or commanded.*	Motivated employees			

The identified subcategories were further organized into categories according to their content, which were then consolidated into overarching main categories. We identified 28 subcategories, seven categories, and three main categories (Table 3). To ensure trustworthiness, the research group discussed several points in the analysis process.

**Table 3:** Main categories and categories.

Main categories	Categories
E‐leadership in nursing is here to stay	• Current status and development opportunities of e‐leadership in nursing
	• Factors leading to e‐leadership in nursing

Lack of consistent e‐leadership practices in nursing	• E‐leadership as an opportunity and challenge for nurse leaders
	• Current organizational structures

E‐leadership is a challenge for nurse leaders and staff	• Key success factors in e‐leadership in nursing
	• E‐leadership from the perspective of healthcare professionals
	• Nurse leaders’ e‐leadership competencies: current status and future needs

### 2.5. Ethical Considerations

The participating organization granted research permission for this study and assessed its ethical considerations. In line with Finnish legislation, the research did not require approval from a research ethics committee, as the interviews did not involve patients or minors, nor did they affect physical or mental integrity [28]. Research approval was obtained from a research organization. The study was conducted and reported with accuracy, honesty, and transparency, in full accordance with established ethical principles. All participants received written information outlining the voluntary nature of their participation and their right to withdraw from the study at any time. They were given the opportunity to discuss the study and ask questions directly with the researchers. Before the interviews, participants were provided with both verbal and written information about the study, its objectives, and data protection practices. Verbal and written consent were obtained from each participant. To maintain anonymity, all personal information was removed from the transcripts.

## 3. Results

The participants (*n* = 14) were frontline NLs, specifically nurse managers, working in a large university hospital in Finland. Their healthcare work experience ranged from 13 to 38 years, with experience in e‐leadership ranging from two to 20 years. Most had 5 years or less of e‐leadership experience. The teams they managed ranged from seven to 70 subordinates. Almost all participants held a master’s degree (Table 4).

**Table 4:** Participants’ characteristics.

Demographic data	N	%
Gender		
Female	14	100
Age (years)		
31–40	3	21.4
41–50	6	42.9
51–60	2	14.3
61–70	3	21.4
Work experience		
Clinical work		
0–5	1	7.1
6–10	1	7.1
11–15	2	14.3
16–20	6	42.9
21–30	4	28.6
Leadership experience		
0–5	7	50.0
6–10	4	28.6
11–15	1	7.1
15–20	1	7.1
21–30	0	0
31–35	1	7.1
E‐leadership experience		
0–5	11	78.6
6–10	2	14.3
11–15	0	0
16–20	1	7.1
Number of subordinates		
0–10	2	14.3
11–20	1	7.1
21–30	6	42.9
31–40	1	7.1
41–50	0	0
51–60	2	14.3
61–70	2	14.3
Highest education		
Bachelor’s degree	2	14.3
Master’s degree	12	85.7

### 3.1. Category I: E‐Leadership in Nursing Is Here to Stay

#### 3.1.1. Current Status and Development Opportunities of E‐Leadership in Nursing

E‐leadership in nursing takes diverse forms depending on the work environment. This study included NLs from psychiatric, somatic, home care, and reserve staff units. NLs often manage multiple geographically dispersed teams, necessitating physical distance from some teams. NLs in hospital units where staff are always on‐site typically have remote workdays, while healthcare professionals may be geographically dispersed or working remotely themselves. NLs emphasized the importance of physical presence and preferred a hybrid leadership model. This model offers both challenges, such as a lack of structure and reduced human interaction, and benefits, including increased efficiency and autonomy.

ID12: *“This hybrid model is definitely a must. I don’t think it’s possible for me to work fully remotely.”*

NLs acknowledge the increasing relevance of e‐leadership due to the expanding scope of their responsibilities and the evolving nature of healthcare work. Despite institutional inertia, many NLs act as change agents, advocating for digital care pathways and new leadership approaches. E‐leadership is one of many changes in healthcare, and NLs are gradually adapting to this new leadership style. The use of technology has increased, aligning nursing with changes seen in other industries. The COVID‐19 pandemic accelerated the shift toward digital services, highlighting the feasibility of remote operations and prompting expectations for further growth in e‐leadership. Artificial intelligence is seen as promising for the future; however, none of the participants had direct experience of using it.

ID6: *“So in a way, this has been one major challenge among many. And now that we’ve managed to get through it to this point, I hope it’s taught each of us something…that when we face other kinds of challenges or changes, we can reflect on them through the lens of this process work. I truly believe this has taught us a lot, especially about our field and leadership. Leadership in particular.”*

E‐leadership has introduced time‐saving opportunities, enabling leadership independent of time and place. Online meetings improve information flow, and uninterrupted workdays reduce commuting. Online education has expanded access to training. However, the boundary between efficiency and overachievement is thin, with long workdays and multitasking becoming common. Some NLs limit remote work due to self‐imposed pressure.

ID5: *“I get a bit hysterical, I even take my phone with me to the bathroom, and it feels like I can’t even take a proper lunch break.”*

Concerns about staff well‐being are heightened in remote settings, where signs of burnout may go unnoticed. To address this, NLs have developed structures such as remote work guidelines and clear divisions of responsibility. Flexibility is valued, but being physically present remains essential in many situations.

ID14: *“Well, maybe with a few employees—and they’re very conscientious by nature. I think behind it there’s this strong sense of needing to help everyone and do everything as perfectly as possible.”*

Communication practices have evolved, with NLs using platforms like Teams, e‐mail, phone, and messaging apps. Teams is the primary tool for meetings, messaging, and document sharing, though its extensive use has also led to information overload. NLs have streamlined communication channels to improve clarity. Meetings vary in format, including live, online, and hybrid, with technology‐related challenges still present to some extent. Some NLs have returned to only holding meetings in person, but that is not feasible for all owing to the area of their responsibilities. Hybrid meetings remain challenging, and camera use is inconsistent, requiring active encouragement to foster engagement and reduce multitasking.

Written communication is central in e‐leadership but can be misinterpreted. NLs strive for clarity and neutrality and sometimes use emojis in informal contexts to help. They often initiate contact with staff, seeking reciprocal communication and feedback.

ID8: *“(Written communication) really is kind of an art form, you know.”*

Data‐driven leadership has improved resource planning and transparency. NLs utilize various data sources to monitor performance, particularly in remote care settings, although some staff members can perceive the practice as intrusive. Further integration of digital platforms is expected to enhance these processes in the future.

E‐leadership and remote work can empower both leaders and staff. Nurses can leverage their competencies through digital services, while leaders can utilize data for knowledge‐based leadership. However, NLs face challenges in managing the overwhelming amount of information and ensuring healthcare professionals are informed. The cognitive load not only is prevalent among staff but also significantly affects NLs. Some interviewees were concerned about the future development of this trend.

Overall, e‐leadership and remote work offer flexibility, better work–life balance, and ecological benefits. They also support part‐time staff and entrepreneurial activities. However, managing cognitive load and maintaining human connection remain ongoing challenges.

ID5 *“This has definitely given a certain kind of freedom not only to the employees but also, in a way, to myself.”*

#### 3.1.2. Factors Leading to E‐Leadership in Nursing

We identified several factors that spurred an increase in e‐leadership in nursing. First, there was the catalytic role of the COVID‐19 pandemic, which proved a critical inflection point. The associated operational restrictions compelled healthcare organizations to rapidly adopt digital leadership practices. NLs, despite limited prior experience, engaged in a process of experiential learning. The crisis demonstrated the feasibility of e‐leadership and accelerated the acquisition of digital competencies among both leaders and staff. Maintaining interpersonal connections under restrictive conditions underscores the importance of hybrid leadership models.

ID13: *“It was kind of like we had no choice but to succeed at that point, but if it had been completely without any face-to-face interaction at all, it probably would’ve been more challenging.”*

The second factor is preexisting hybrid practices and technological expansion. Prior to the pandemic, hybrid work models were already in use, particularly among NLs managing distributed teams. However, these practices were limited to basic tools such as phones and laptops. The pandemic expanded the technological landscape, introducing new platforms that enhanced the scope and visibility of e‐leadership.

Third, the expanding scope of leadership responsibilities, the increasing size and complexity of NLs’ areas of responsibility, and the growing number of employees, often spanning multiple teams and locations, necessitate e‐leadership. It is no longer feasible to maintain a physical presence in all units simultaneously, making digital coordination essential.

Fourth, e‐leadership and remote work are cost‐effective for both organizations and individuals. They reduce commuting time and expenses, optimize working hours, and lower sick‐leave costs by enabling work‐from‐home arrangements during minor injuries or ailments. Additionally, hospital office spaces have become overcrowded and noisy, prompting a preference for remote working among NLs and staff.

ID14: *“And then my workspace is this little windowless cubicle, it’s lovely to work remotely every now and then.”*

Fifth is information overload and cognitive load management. The digitalization of healthcare has led to an overwhelming volume of information. Initially, the focus was on mastering new platforms; now, the challenge lies in managing, organizing, and retrieving information efficiently. NLs have responded by establishing internal structures and promoting self‐guidance among employees.

ID5: *“What really annoys me the most are those kinds of people who don’t show up to the meetings we’ve agreed on, and then afterwards complain that they weren’t informed. I mean, honestly, that’s totally their own fault. The opportunities are there, and you have to take part. I really emphasize personal responsibility in those cases.”*

Finally, remote workdays offer NLs uninterrupted time for tasks requiring concentration, such as strategic planning, ideation, and creative problem‐solving. In contrast, hospital environments are characterized by frequent interruptions, limiting opportunities for deep work.

ID9: *“I see it like this: that’s also why I take remote workdays…because if I have something that really requires deep focus, it works better when I’m at home than here, where people sometimes drop by my office.”*

### 3.2. Category II: Lack of Consistent E‐Leadership Practices in Nursing

#### 3.2.1. E‐Leadership as an Opportunity and Challenge for NLs

NLs generally approach e‐leadership and remote work with curiosity and optimism, grounded in trust in their colleagues’ professionalism. For some, the opportunity to lead digitally has been a key motivator in their career choices. While many value the flexibility of remote work, others still prefer physical presence in clinical settings, recognizing that effective leadership often requires a hybrid approach. In some cases, the demands of e‐leadership have even influenced job changes.

ID2: *“I was actually interested in learning and growing myself into this kind of e-leadership role within this unit.”*

Digitalization has transformed networking and project management, with most interactions now occurring in hybrid or fully remote formats. NLs have adapted to being led remotely themselves, viewing it as a learning opportunity. Support from chief nurses has encouraged the integration of remote workdays, which are associated with improved well‐being, reduced commuting, and increased autonomy. However, NLs must manage their own boundaries, ensuring they have sufficient rest time, allocate time for physical activity, and avoid overcommitment.

Despite high job satisfaction, NLs face challenges in maintaining a work–life balance. Many monitor notifications outside working hours and report a tendency to work while ill. Remote workdays often lack natural breaks, which can contribute to sustained hypervigilance and difficulty disengaging. While NLs recognize this issue, they struggle to implement effective solutions.

ID8: *“Yeah, I do shut down my computer and try to keep my phone on silent, but…I guess I’m a bit too conscientious.”*

Most NLs identify with a coaching leadership style, emphasizing psychological safety, individualized support, and team engagement. Although they perceive few qualitative differences between in‐person and e‐leadership, virtual environments require more deliberate efforts to build trust and legitimacy. Maintaining relational depth and engagement is particularly challenging if staff members view remote work as somehow less legitimate than office‐based work.

ID12 *“I actually see e-leadership and in-person leadership as exactly the same thing.”*

E‐leadership demands creativity, interdisciplinary competence, and innovation. NLs support involving healthcare professionals in shaping future practices and envisage their organizations as pioneers in digital leadership. Nearly all leadership tasks, including sensitive conversations and evaluations, can be conducted remotely, preferably using cameras to maintain presence.

ID14: *“But maybe there’s a certain kind of creativity that comes with digital work, like having the courage to think outside the box and try out new things. So maybe we need a bit more of that same creativity in our leadership systems, too.”*

Maintaining availability and approachability is essential, regardless of physical location. While remote work reduces spontaneous contact, proactive communication and clear scheduling help manage expectations. In units with staff on‐site, physical presence remains important. Diminished contact with colleagues during remote days can jeopardize the sense of professional competence, especially among less experienced NLs, and feelings of inadequacy may arise.

A recurring concern is the erosion of informal interaction and community in remote settings. Task‐oriented communication may weaken interpersonal relationships and delay the recognition of issues such as burnout or substance misuse. Providing feedback is also more complex without direct observation, and doing so requires vigilance and sensitivity.

ID2: *“Sometimes I do wonder. Do I really know how my employee is doing? How they’re feeling? If everything’s okay with them?”*

Digital communication practices have improved, and there are fewer instances of inappropriate behavior on platforms like Teams. Nonetheless, NLs occasionally need to moderate discussions and clarify expectations to maintain respectful dialogue.

Ethical challenges also emerge, particularly in assessing employee well‐being equitably when visibility varies. While remote work opportunities for nursing staff are limited, perceptions of fairness in workload distribution must be actively addressed. Transparency and open communication are key to preventing remote work from being seen as a privilege.

ID2: *“Lately, we’ve been seeing a bit of a side effect where the perspective has kind of shifted. Remote work is increasingly being seen as an individual benefit, rather than something shaped from the standpoint of the whole work community.”*

Ultimately, e‐leadership fosters delegation, transparency, and flexibility but also intensifies multitasking and cognitive load. Some NLs reported issues with underperformance and isolation can be issues, particularly among staff who prefer extensive remote work arrangements. NLs must balance these dynamics by providing empathy and structure, utilizing performance indicators, and applying strategic oversight.

#### 3.2.2. Current Organizational Structures

Despite following guidelines for remote work, including data protection instructions and performance level obligations, the employer of the interviewed NLs had no formal arrangements governing e‐leadership. Some NLs desire indicative guidelines and solution models, though overly strict guidelines could compromise flexibility. NLs would appreciate e‐leadership training provided by the organization since experiences of e‐leadership vary across divisions, with different attitudes from nurse directors. NLs desire more innovation in leadership practices and a consensus on e‐leadership, recognizing the permanence of digitalization.

ID7: *“I feel like people have been doing that quite a bit within the operational units. For instance, supervisors reportedly work remotely as often as once a week.”*

Differences in remote work practices exist among professional groups. Physicians often dictate their work conditions and may work mainly remotely if it suits their job responsibilities. Department secretaries also frequently work remotely. NLs seek more decision‐making power and influence for themselves and nurses concerning remote work and e‐leadership. Remote work has become an attractive factor and a recruitment asset in nursing, appealing to both nurses and NLs.

Working conditions in remote settings vary and are largely the responsibility of NLs or staff members. Confidentiality must be maintained. NLs have typically set up a workstation, but many work at dining tables or elsewhere. The organizations provide laptops, smartphones, and headphones, although ergonomics are not always ideal in home offices, despite the familiarity. NLs are sometimes concerned about the safety of employees because they cannot directly monitor their activities.

Patients became accustomed to remote services during the COVID‐19 pandemic and often prefer them when feasible. The means available to patients through which to contact healthcare professionals are increasing. The convenience has led to some overuse of services, presenting a new challenge for the healthcare system. NLs utilize patient feedback, a quality indicator, to monitor the quality of nursing care.

ID12: *“These things do come up. Nowadays, patients are quite quick to give feedback about whether they want to meet remotely or not. Some definitely prefer it, while others don’t want it at all.”*

### 3.3. Category III: E‐Leadership Is a Challenge for NLs and Staff

#### 3.3.1. Key Success Factors in E‐Leadership in Nursing

While trust is vital in any leadership setting, it is particularly important in an e‐leadership scenario. Trust is fundamental to successful e‐leadership in nursing. Unlike traditional nursing leadership, where NLs are physically present and can monitor daily activities, e‐leadership relies heavily on trust. NLs must assume that employees are trustworthy and diligent. Over time, many NLs have learned to trust their teams at a deeper level and value the trust they receive from their chief nurses. Building mutual connections with employees is crucial for fostering trust. Key behaviors NLs identified as important for building trust included being available, flexible, a role model, listening to employees, and maintaining a positive and encouraging attitude. It takes time to create trust and often requires a physical presence. Achieving a state of mutual trust is highly satisfying for NLs, especially when it requires effort. NLs appreciate when employees seek their help, viewing it as demonstrating trust. However, trust can be lost, often when employees fail to fulfill their responsibilities. Addressing trust‐related issues is challenging, but it is necessary.

ID14: *“Well, yes, it really starts from there, like I said, those first. I think it all begins with recruitment. I always try to make sure those moments are unhurried and meaningful, good encounters. Because if something goes wrong at that stage, it tends to carry on. So yes, the beginning is really important.”*

Clear goal setting and communication are essential elements of building trust. Job descriptions must be precise, and overlapping responsibilities between professional groups should be addressed. Role responsibilities and goals should be clearly stated during recruitment to avoid misunderstandings.

E‐leadership and remote work demand self‐control and self‐management skills from both NLs and employees. Employees need to strengthen those skills to adapt to changing situations, applications, and platforms. For NLs, this involves practicing delegation and prioritization. Motivated employees generally meet new requirements effectively.

Transparency is crucial to avoid misunderstandings. Uncertainty about remote work content has led to negative attitudes among colleagues. Remote working in the nursing context encompasses a diverse range of services, including telephone consultations, remote receptions, patient communication via chat platforms, and delivering care through digital treatment paths and therapies.

Some physical presence is essential for successful e‐leadership in nursing. A lack of presence can amplify negative perceptions, and straightforward communication can be particularly challenging. NLs strive to meet their teams regularly, although that depends on the unit’s environment. Meeting the entire team can be challenging, especially in shift work and remote work settings. NLs prefer handling certain matters in person to avoid misinterpretation and to be able to observe nonverbal cues. Such matters typically include professional development, questions of ability and disciplinary issues, feedback, job interviews, and onboarding. Being familiar with their staff allows NLs to handle some situations online later.

ID5: *“But it’s also something that kind of just happens…you don’t even really notice it. Like, if you don’t see someone regularly, it’s not always that easy to bring things up in a natural, straightforward way.”*

Meeting in person helps build relationships, particularly during the onboarding process. NLs are concerned about the effects on community spirit and cooperation skills if remote working becomes excessive. They emphasize the importance of personal contact in any leadership style. Nursing is demanding, and NLs aim to support employees’ success, especially during times of change. NLs’ presence can stabilize the work environment during crises or disruptions to the routine. Some NLs were concerned about a loss of control when their role was predominantly delivered via e‐leadership and stressed the need to balance trust with the need for supervision.

#### 3.3.2. E‐Leadership From the Perspective of Healthcare Professionals

Healthcare professionals generally have a positive view of working remotely, as it allows for a flexible combination of work and personal life. However, not all nurses prefer or are able to work remotely due to confidentiality requirements or unsuitable home working conditions. Even when remote work is not wholly feasible, certain tasks, such as online training or specific responsibilities, can be performed from home. Deputy nurse managers also occasionally work remotely, planning tasks that require deep concentration.

The extent of remote work varies among nurses. Some units allow nurses to work remotely three to 4 days a week, particularly for tasks like phone assessments or digital therapy. However, a complete remote work schedule is rare and typically reserved for special cases, such as work capacity support.

Technical proficiency is essential for remote work, but nurses’ skills vary widely. While some find basic tasks, such as reading e‐mails, challenging, others are more adept with technology. Initially, most staff were unfamiliar with platforms like Teams and online meetings felt awkward, especially with regard to camera and microphone use. Over time, practice has improved comfort levels, though clinical nurses still find it challenging. NLs encourage training in digital skills and provide guidance on using digital tools.

NLs often consider how to provide sufficient support for remote employees, striking a balance between trust and the need for employees to seek help when necessary. They remind employees to take breaks and care for themselves. Employees can also access ergonomics counseling for the remote setting. Some NLs believe they can support employees more effectively remotely, as they can connect with them regardless of physical location. They have observed significant improvements in some employees’ work ability brought about by the remote work option.

ID11: *“Yeah, actually, e-leadership offers quite a lot of possibilities. For one, it means I can always provide support, no matter where I am physically.”*

Monitoring and balancing workloads is another aspect of NL support. They aim to dispel the notion that NLs are the sole point of contact for unclear situations, promoting the utilization of multidisciplinary teams. Peer support is also encouraged, with regular meetings and designated mentors to strengthen support networks. Teams’ channels facilitate easy communication and technical advice among colleagues.

Generational differences in attitudes and skills are evident, with younger employees more comfortable with technology due to their educational experiences. Older employees may view nursing as inherently tied to hospital settings. NLs strive to encourage employees and express gratitude whenever possible, fostering a supportive work environment.

ID9: *“Young people tend to understand and are quite used to this kind of e-leadership model, whereas those who are a bit older, in terms of working age, are more accustomed to a different way of working. And with them, you can see a certain dissatisfaction with the lack of face-to-face interaction.”*

#### 3.3.3. NLs’ E‐Leadership Competencies: Current Status and Future Needs

Most of the NLs interviewed had no formal e‐leadership training and expressed a desire for training in methods and best practices. Generally, NLs have developed their e‐leadership skills through self‐teaching and experiential learning. While technical training and digitalization are important, they do not necessarily enhance e‐leadership competencies. NLs find technical aspects to be the easiest part of e‐leadership and also see a need for employee training. Only one interviewee had formal e‐leadership training, having recently completed a master’s degree.

Some educational programs include e‐leadership, but the content often focuses on communication and may be difficult to apply in nursing. NLs recognize the importance of strong technical skills and hope for future training specifically tailored to e‐leadership in the nursing context that reflects the expanding role of e‐leadership and digital services. NLs would also appreciate peer support, mentoring, and clear, uniform guidelines from their organizations. Additionally, they would need to allocate time for training, as their current schedules often do not allow for participation in training courses.

ID2: *“I don’t mean it has to be some long, multi-hour, or credit-based course. Just something that would provide some practical tools from a leadership perspective.”*

## 4. Discussion

Our study discusses the current state and development needs of e‐leadership in nursing. It highlights the importance of trust, presence, and availability, as well as clear guidelines, training needs, and the potential for future growth. The COVID‐19 pandemic was a pivotal moment for e‐leadership in nursing, compelling healthcare organizations to rethink their care and leadership methods. This shift was unprecedented, with NLs recalling it as a period of learning by doing, given their limited experience in e‐leadership. Despite the initial challenges, the pandemic demonstrated the feasibility and benefits of e‐leadership, requiring trust, clear communication, and technical skills.

E‐leadership in nursing is a multifaceted phenomenon shaped by diverse organizational contexts, evolving work practices, and the increasing integration of digital technologies. NLs’ leadership is characterized by a hybrid model that blends physical presence with remote engagement, reflecting both the necessity and limitations of e‐leadership in clinical environments. Despite the growing reliance on e‐leadership, NLs emphasize the irreplaceable value of face‐to‐face interaction. The hybrid model offers efficiency and autonomy but also introduces challenges related to interpersonal connection, organizational structures, and role clarity. Ameel et al. [9] emphasized similar aspects of nursing leadership, advocating for a hybrid leadership model in the future. In this study, NLs with greater e‐leadership experience seem to have more robust guidelines and standards with their teams than the NLs with more superficial e‐leadership experiences.

Despite reporting high levels of job satisfaction, NLs face an increased cognitive load, frequent multitasking and blurred work–life boundaries. Recent studies have demonstrated that cognitive overload among nurses affects their mental, physical, and emotional well‐being [29]. There is a need for targeted interventions to manage workload and cognitive load [30]. NLs express concern over employee well‐being, particularly the risk of unnoticed burnout among conscientious staff. E‐leadership and remote work require strong self‐management skills. These results strengthen previous evidence [1]. It is clear that NLs must balance the benefits of digitalization with the risks of multitasking and reduced interpersonal connection. Additionally, NLs must navigate multiple platforms and devices, which can lead to information overload, highlighting the need for stronger self‐regulation and organizational support.

Trust and virtual presence are critical components of e‐leadership. Similar observations were reported by Cowan [7] and Sharpp et al. [11]. Unlike traditional nursing leadership, where NLs are physically present and can monitor daily activities, e‐leadership relies heavily on trust. NL behavior identified as important for building trust includes being available, flexible, a role model, listening to colleagues, and maintaining a positive and encouraging attitude. NLs often struggle with feelings of inadequacy related to their availability and flagged the need for some level of monitoring and supervision, which is common in healthcare organizations [31].

As healthcare organizations increasingly adopt remote and hybrid models, NLs are navigating a complex leadership landscape that demands both technological competence and relational sensitivity. It is crucial that NLs receive training focused specifically on e‐leadership methods and best practices. Training should expressly address the digital, interpersonal, and ethical complexities of leading care professionals remotely. Most NLs have developed their e‐leadership skills through self‐teaching and experiential learning, highlighting the need for formal training programs. These needs have yet to be addressed, despite frequent reporting in studies [9, 15, 17, 32]. E‐leadership in nursing has shifted over time from learning to use platforms, and today, NLs must manage huge volumes of information and ensure effective communication.

Avolio et al. [12] cautioned against viewing e‐leadership merely as an extension of traditional leadership, as it encompasses distinct features and challenges. This study reveals a partial discrepancy, as the NLs interviewed recognized e‐leadership as a current form of leadership but also depicted it as particularly challenging, especially in terms of interpersonal relationships, giving feedback, identifying situations requiring intervention, and maintaining fairness. Avolio et al. [12] recognized the importance of context in e‐leadership, and our findings also indicate that contextual factors shape e‐leadership practices. The NLs informing this study had developed diverse approaches tailored to their specific operating environments.

NLs face different demands from employers than other employees. Simultaneous extensions to the scope of their work and the requirement to be continuously accessible and responsive can, for some NLs, result in a perceived sense of inadequacy. Work well‐being often deteriorates in remote work environments and in e‐leadership, where the focus is predominantly on performance, and breaks are seldom taken. Over time, NLs have come to value their breaks (even if it is only a lunch break) and encourage employees to take regular breaks. This issue remains a concern and warrants ongoing monitoring. NLs frequently work beyond regular hours, yet do not perceive this as alarming.

The future of e‐leadership in nursing seems promising given the potential for further integration of technology and digital services. NLs have identified these opportunities, although their healthcare organization employer does not yet offer sufficient support, as earlier research also noted [8]. NLs often develop their own frameworks for e‐leadership and communication, but the absence of standardized policies can lead to inconsistency and perceptions of unfairness. NLs hope for more training opportunities, peer support, and clear guidelines from their organization.

As technology evolves, e‐leadership can offer even more opportunities for NLs and team members, enhancing efficiency, transparency, and data‐driven decision‐making. Given the scarcity of healthcare resources, organizations should leverage e‐leadership and remote work opportunities in the future.

### 4.1. Limitations

The criteria of trustworthiness in a qualitative study include credibility, transferability, dependability, and confirmability [33]. Credibility was enhanced by interviewing NLs with experience in e‐leadership who were willing to share their insights. Inclusion criteria and purposeful recruitment facilitated the identification and approach of NLs within a large healthcare organization in southern Finland. The NLs came from various units and hospital environments. A researcher on a doctoral‐degree program conducted the interviews. The participants are described, the results present authentic quotations, and an example of the analysis process is provided, enhancing the study’s credibility and transferability. Dependability is ensured by detailing the data collection and analysis processes. Furthermore, confirmability is enhanced through ongoing reflection, self‐awareness, and continuous discussions with the research team throughout the process. These factors strengthen the study.

There are also limitations. This research is a qualitative study, and its results reflect the experiences of 14 NLs and are not generalizable to the whole domain of nursing e‐leadership. Finland’s high level of digitalization [34], including digital patient pathways [35], supports remote work and e‐leadership. That should be borne in mind when comparing the results to those in other environments. However, increasing the awareness of e‐leadership in nursing to leverage appropriate opportunities is important. The current findings could support transferability to other organizations in the future if there were quantitative data derived from larger samples. Future studies should take that into account.

### 4.2. Implications for Nursing Management

• Setting clear goals and job descriptions for teams and individual nurses is essential.
• For NLs heavily involved in e‐leadership, establishing a reasonable number of communication channels is beneficial.
• If the work involves frequent online meetings, the team should agree on rules for camera and microphone use.
• Transparency regarding the content and amount of remote work is important for fostering mutual trust between nurses and NLs.
• Regular in‐person meetings are indispensable for fostering community spirit and building interpersonal relationships.
• Organizations should provide indicative guidelines and appropriate training for e‐leadership.
• Organizations need to ensure the same rules for different professionals.

### 4.3. Future Research

The phenomenon of cognitive overload was highlighted in the results of our study. While research has been conducted on cognitive overload among nurses, there is a notable gap in the literature regarding cognitive overload experienced by NLs. Given that NLs are expected to alleviate the cognitive burden on nurses, it is crucial to understand how NLs themselves manage cognitive overload.

E‐leadership in nursing should be examined across various management levels. NLs are increasingly networking online and often describe chief NLs as “e‐leaders”. Therefore, a comparative study across different organizational levels could provide valuable insights. Additionally, while expectations are being cemented on the operational level, the organizational aspect of e‐leadership remains underexplored.

NLs reported that a hybrid work model is the most suitable for the healthcare environment, with remote work being particularly beneficial for tasks requiring deep concentration. Future research should explore how work well‐being can be enhanced, for example, by determining the optimal ratio of remote workdays for NLs.

## 5. Conclusions

In conclusion, this study strengthens previous findings on and provides new insights into the current state of e‐leadership in nursing. E‐leadership in nursing is a dynamic and evolving practice that requires a delicate balance between digital efficiency and human connection. While e‐leadership presents several challenges, it also offers significant benefits and opportunities for the nursing profession, enabling flexibility in the work–life balance. What started as a necessity has become an attractive factor and a valuable recruitment asset in nursing. However, e‐leadership also requires strong self‐control and self‐management skills from both NLs and employees. Motivated employees generally meet new requirements effectively, but there is a need for continuous development of these skills. As healthcare continues to digitalize, NLs must be equipped with the tools, training, and organizational support necessary to lead confidently and compassionately in both virtual and physical spaces, as e‐leadership does present attitudinal challenges. E‐leadership in nursing is not merely a digital extension of traditional leadership; it requires a rethinking of leadership practices to align with the realities of modern, hybrid healthcare environments.

## Conflicts of Interest

The authors declare no conflicts of interest.

## Funding

This work did not receive any funding. Open access publishing facilitated by Vaasan yliopisto, as part of the Wiley ‐ FinELib agreement.

## Supporting Information

Additional supporting information can be found online in the Supporting Information section.

## Supporting information


**Supporting Information 1** 1. The Standards for Reporting Qualitative Research (SRQR) checklist [20], which is used to guide reporting, is presented in the supporting information.

**Supporting Information 2** 2. Interview guide.

## Data Availability

Research data are not shared.
