# Supplementary material for: Nurse Leaders’ Perceptions and Practices of E‐Leadership: A Qualitative Study
Source: J Nurs Manag. 2026 Jan 8;2026:2583936. doi: 10.1155/jonm/2583936 (PMC12780739; doi:10.1155/jonm/2583936)
Supplement: Supplementary file 2 — Supporting Information 2 2. Interview guide. [file JONM-2026-2583936-s001.docx]

Supplement: Interview guide

Background Information

- Job title / position
- Number of subordinates
- Nature of the work unit
- Age
- Gender
- Educational background
- Work experience in years:
  - Clinical work
  - Supervisory/managerial work
  - E-leadership

Start of Interview:

Opening Question:
*What kind of experience do you have in e-leadership?*

| Theme | Details of the theme | Relevant literature example |
| --- | --- | --- |
| Theme 1: Organizational Structures and Competence | Discussion focuses on organizational support and opportunities for e-leadership.  *Example question: What organizational guidelines do you have for e-leadership?* | Terkamo-Moisio, A., Karki, S., Kangasniemi, M., Lammintakanen, J., & Häggman-Laitila, A. (2022). Towards remote leadership in health care: Lessons learned from an integrative review. *Journal of Advanced Nursing*, *78*(3), 595–608. https://doi.org/10.1111/jan.15028 |
| Theme 2: Social Relationships | Relationships with employees and building trust.  *Example question: How do you maintain relationships with employees remotely?"* | Kiljunen, M., Laukka, E., Koskela, T. K., & Kanste, O. I. (2022). Remote leadership in health care: A scoping review. *Leadership in Health Services (1751-1879)*, *35*(1), 98–115. CINAHL. https://doi.org/10.1108/LHS-06-2021-0059 |
| Theme 3: Well-being at Work | Own and employees’ well-being from the perspective of e-leadership.  *Example question: How do you take care of your own well-being when working remotely?* | Hurmekoski, M., Häggman-Laitila, A., Lammintakanen, J., & Terkamo-Moisio, A. (2023). Nurse leaders’ experiences of remote leadership in health care. *Leadership in Health Services (Bradford, England)*, *ahead-of-print*(ahead-of-print), 579–594. https://doi.org/10.1108/LHS-01-2023-0003 |
| Theme 4: Post-Pandemic Period *(if not addressed in other themes)* | Discussion on leadership during the pandemic and the evolved practices of e-leadership.  *Example question: If the participant has leadership experience during the pandemic: how has e-leadership evolved past-pandemic?* | Numanovic, V., Jalonen, H., Lindell, J., & Jacobsson, J. (2024). E-leadership in nursing – a systematic review. *Finnish Journal of eHealth and eWelfare*, *16*(1), Article 1. https://doi.org/10.23996/fjhw.137575 |
| Theme 5: The Future | Future perspectives on e-leadership in nursing management.  *Example question: What kind of developments do you anticipate in the future?* | Ameel, M., Myllynen, M., & Kallakorpi, S. (2022). Exploring Hybrid Leadership: Experiences of Remote Leadership in Healthcare. *JONA: The Journal of Nursing Administration*, *52*(12), 653–658. https://doi.org/10.1097/NNA.0000000000001227 |
